# Group therapy for health care workers in a general hospital during the COVID-19 pandemic

**DOI:** 10.1192/j.eurpsy.2021.1791

**Published:** 2021-08-13

**Authors:** S. De Miguel-Gimeno, M. Martínez-Roig, S. Margolles-Gareta

**Affiliations:** Psychiatry, Hospital Royo Villanova, Zaragoza, Spain

**Keywords:** group therapy, COVID-19, burn out

## Abstract

**Introduction:**

A considerable percentage of Health Care Workers (HCW) have experienced psychological distress during the COVID-19 pandemic. Data from previous pandemics suggest that HCW might develop psychiatric disorders. Psychosocial and workplace measures can improve mental wellbeing of the MHW. As part of the program of the Hospital to give support to the HCW, five support weekly open dynamic groups have been carried out with HCW from the COVID Areas of our Hospital including the ICU

**Objectives:**

Identify recurrent contents in the group that express areas ofconcern Identify HCW in risk of develop a psuchiatric disorder and refer them to their apropiate level o

**Methods:**

The sessions were carried out in a freely open group and the contents expressed in the sessions were recorded and analyzed ina narrative way. Special attention was given to the the more stresfull activities identified, to Signs of overload and to the ability to seek relief, as well as signs of disruption of personal life outside of work. Four sessions of 90 minutes, with staff of the same area were established and after these four sessions booster sessions was offered through continuity groups to members with need of more long term care as well as individual care.

**Results:**

The recurrent areas identified were Concern about inadequite Personal Protective Equipment Concern about spreading the infection in their own families Need for relief and avoid double turn Uncertainty about the course of the illness Exposure to patients suffering and dying

**Conclusions:**

HCW need nor only psychological support but also pragmatic measures

**Disclosure:**

No significant relationships.

